# Clinical and sociodemographic variables associated with diabetes-related distress in patients with type 2 *diabetes mellitus*

**DOI:** 10.1590/S1679-45082016AO3709

**Published:** 2016

**Authors:** Flávia Cristina Zanchetta, Danilo Donizetti Trevisan, Priscila Peruzzo Apolinario, Juliana Bastoni da Silva, Maria Helena de Melo Lima

**Affiliations:** 1 Universidade Estadual de Campinas, Campinas, SP, Brazil

**Keywords:** Diabetes mellitus, type 2, Stress, psychological, Diabetes complications, Public health nursing

## Abstract

**Objective:**

To evaluate the relation between diabetes-related distress and the clinical and sociodemographic characteristics of type 2 *diabetes mellitus* patients.

**Methods:**

A cross-sectional study based on a secondary analysis of data collected at a specialized care outpatient center in Brazil. Participants completed a questionnaire on sociodemographic and clinical characteristics and the Brazilian version of the Diabetes Distress Scale (B-DDS).

**Results:**

About 31% of the 130 eligible patients reported diabetes distress, and the mean B-DDS score was 2.6. Multiple regression analysis showed the B-DDS score was positively correlated with marital status (p=0.0230), use of diet and physical activities for diabetes management (p=0.0180), and use of insulin therapy (p=0.0030). The “emotional burden”, “regimen-related distress”, and “interpersonal distress” domains from B-DDS were associated with the use of insulin therapy (p=0.0010), marital status (p=0.0110), and the presence of three or more comorbidities (p=0.0175).

**Conclusion:**

These findings suggest the clinical and sociodemographic variables are relatively weak predictors of diabetes-related distress. The highest scores in the B-DDS were observed in the emotional burden domain, indicating the presence of diabetes distress among the participants of the study.

## INTRODUCTION

Today, the non-communicable chronic diseases represent an important public health problem worldwide, since they are associated with high rates of morbidity and mortality and high costs to public health systems.^(1,2)^ Type 2 *diabetes mellitus* (DM2) gets attention because of its chronic nature and high incidence worldwide. The International Diabetes Federation (IDF) reported there were approximately 382 million people with DM2 in 2013 all over the world, and that this number was expected to increase to 592 million by 2035.^(3)^

The objective of DM2 treatment should be to achieve adequate metabolic control, thus preventing long-term chronic complications, which are the main cause of mortality among these individuals.^(1–3)^ DM2 is a complex condition, and effective management depends on self-care activities and on patient's barriers to implement capillary glucose monitoring and insulin therapy.^(4,5)^

Self-management of DM2, patients' concerns about their health condition, and the possibility of developing complications may lead to emotional stress, a condition referred to as diabetes-related distress.^(6,7)^ This is defined as an emotional reaction to the various situations the patient must deal with on an everyday basis, which may have a temporary or permanent negative impact, in the form of negative feelings, such as irritability, sadness, and fear related to the difficulty in controlling the disease.^(6,8,9)^

Diabetes-related distress may have a significant influence on glycemic control. Distress may act directly to deregulate stressor hormones or indirectly, as a higher emotional burden reduces compliance with *diabetes mellitus* (DM) treatment regimes.^(6,10,11)^ This means that patients may be exposed to a higher risk of hyperglycemia, and a poor glycemic control may lead to severe complications and development of comorbidities.

Diabetes-related distress is often confused with depression, but although it is closely related to depression, it is important to distinguish between them if adequate treatment is to be provided.^(6)^ It has been reported that nearly a quarter of all individuals diagnosed with DM2 suffer from depressive symptoms or emotional stress related to DM2, and about 18 to 45% of DM2 patients are diagnosed with diabetes-related distress.^(1,12,13)^ Both depression and diabetes-related distress may interfere with glycemic control and result in elevated glycated hemoglobin A1c (HbA1c) levels. Some studies indicate that diabetes-related distress is a better predictor of hyperglycemia than depression, but evidence is not clear.^(14,15)^ Diabetes-related distress is also considered a risk factor for depression, which is associated with increased morbidity and mortality.^(16)^ Behavioral interventions showed to be promising as a means of enabling patients to manage the emotional burden inherent to DM2, hence improving psychological well-being and diabetes-related health outcomes.^(17)^

The American Diabetes Association recommends routine monitoring of diabetics for psychological problems, such as diabetes-related distress.^(1)^ Emotional well-being is important in managing diabetes, because social and psychological problems may impair the ability of the patient and his or her family to treat the DM2, thus having greater negative influence on health status.^(18–20)^ Clinicians treating DM2 patients should, therefore, monitor patients' psychosocial status so that they can offer interventions as necessary.

Healthcare professionals should have a good understanding of diabetes-related distress to be capable of recognizing the condition and offering affected patients better support so that they are better able to cope with their disease.

## OBJECTIVE

To evaluate the relation between diabetes-related distress and the clinical and sociodemographic characteristics of type 2 *diabetes mellitus* patients.

## METHODS

### Study design and patient selection

This cross-sectional study was undertaken in an outpatient center specialized in DM, arterial hypertension, and obesity at a teaching hospital in the city of São Paulo (SP), Brazil. The outpatient clinic and provides services to patients with complex needs, seen by the *Sistema Único de Saúde* (SUS) [Brazilian Unified Health System]. The participants were recruited consecutively between May and October 2012. The sample comprised 140 men and women, aged 18 years or over, who had been diagnosed as DM2 at least 1 year before, and were on oral antidiabetic agents and/or insulin. The participants were required to have sufficient verbal skills to answer the questionnaires. Exclusion criteria were patients on hemodialysis or with amaurosis.

### Data collection

Data were collected through individual interviews, which took place in a private environment. Social and demographic data (age, sex, schooling level, marital status, employment situation, and individual monthly income), and clinical data (time since diagnosis, comorbidities, treatment, and HbA1c level) were gathered. Later, the Brazilian version of the Diabetes Distress Scale (B-DDS) was applied.

### Measurements

The Diabetes Distress Scale (DDS) was developed in the United States, in 2005, from three other scales: the Measurement of Emotional Adjustment in Diabetic Patients (ATT39), the Questionnaire on Stress in Patients with Diabetes--Revised (QSPD-R) and the Problem Areas in Diabetes (PAID) scale.^(7)^ A Brazilian version of DDS was produced in 2011 and validated in 2015.^(21,22)^ The final Brazilian version consists of 17 items, divided into four subscales: emotional burden (5 items), physician-related distress (4 items), regimen-related distress (5 items) and interpersonal distress (3 items).

Patients responded the items on the B-DDS using a 6-point Likert scale ranging from 1 (non-problem) to 6 (serious problem), to indicate how much diabetes-related stress they had experienced over the previous month. Item scores are averaged to a total score between 1 and 6, thus higher values indicate greater distress and subscale scores are the averages of scores for the items making up that subscale. Scores of 3 or more indicate that the respondent is suffering from diabetes-related distress. Total scores of 3 or higher are considered to reflect clinically meaningful distress levels.^(7,21,22)^

### Statistical analysis

Qualitative variables were reported as frequencies and percentages, and descriptive statistics (mean and standard deviation) were calculated. We also prepared five multiple linear regression models, with B-DDS subscale scores as dependent variables, and the other investigated variables as independent ones.^(23)^ In these models, the Stepwise criterion was applied, as well as the Box-Cox transformation with the dependent variables. We calculated regression coefficients, the related confidence intervals and p values. We also calculated the R^2^ coefficient value for each of the adjusted models. Cohen suggested the following criteria for evaluating R^2^: 0.1 to 0.29 as weak; 0.3 to 0.49 as moderate; >0.5 as strong.^(24)^ A significance level of 5% was applied to all tests. Analyses were carried out with Statistical Analysis System (SAS) statistical software, version 9.4.

### Ethical considerations

The participants were informed as to the objective, procedures, risks and benefits of this study. This project was approved by the local Ethics Committee, under protocols number 1.169.686, and CAAE: 46860015.8.0000.5404; all participants signed a written Informed Consent Form. Our study complies with the Declaration of Helsinki on medical research involving human subjects.

## RESULTS

A total of 140 DM2 were recruited. Ten patients were excluded after preliminary data analysis because of missing values; hence, the final sample had 130 patients. The sociodemographic and clinical profile of the sample is presented on table 1. More than half the participants were at least 60 years old (52.3%), living with a partner (66.9%), had 4 years or less of schooling (64.6%), and had a monthly income of less than two minimum wages (41.5%). A large majority of the sample (70.8%) had had DM2 for more than 5 years, 55.4% were on insulin, and 55.4% had three or more comorbidities. Just over three quarters of the participants (76.9%) reported that they engaged in regular physical exercise and followed a healthy diet.

**Table 1:** Social, demographic, and clinical characteristics of patients

Sociodemographic and clinical variables		n (%)
Sex		
	Female	72 (55.4)
	Male	58 (44.6)
Age, years		
	Mean (SD)	60.4 (9.4)
	Individuals <60	62 (47.7)
	Individuals ≥60	68 (52.3)
Marital status		
	Without a life partner	43 (33.1)
	With a life partner	87 (66.9)
Schooling level, years		
	≤4	84 (64.6)
	5–8	18 (13.9)
	≥9	28 (21.5)
Employment status		
	Active	48 (36.9)
	Retired	61 (46.9)
	Non active	21 (16.2)
Household income (number of minimum monthly wages)*		
	Mean (SD)	3.1 (2.4)
	≤2	54 (41.5)
	>2 e ≤4	41 (31.5)
	>4	35 (27.0)
Time since diagnosis, years		
	≤5	38 (29.2)
	>5	92 (70.8)
HbA1C		
	Mean (SD)	8.6 (1.9)
	<7	31 (23.8)
	≥7	99 (76.2)
Comorbidities		
	<3	58 (44.6)
	≥3	72 (55.4)
BMI		
	Normal	30 (23.1)
	Overweight	45 (34.6)
	Obese	55 (42.3)
Use of diet and physical exercise to manage disease (self-reported)		
	No	30 (23.1)
	Yes	100 (76.9)
Use of oral antidiabetic agents		
	No	22 (16.9)
	Yes	108 (83.1)
Use of insulin		
	No	58 (44.6)
	Yes	72 (55.4)
Number of medicines taken		
	<5	28 (21.5)
	≥5	102 (78.5)
Experienced a hypoglycemic episode during the last month		
	No	81 (62.3)
	Yes	49 (37.7)

* US$ 300.00.

SD: standard deviation; BMI: body mass index; HbA1C: hemoglobin A1c.

### Brazilian version of the Diabetes Distress Scale and subscales

The prevalence of diabetes-related distress (B-DDS score ≥3) was 31.5%. The mean total B-DDS score was 2.6 (standard deviation 1.07); the domain that attracted the highest mean score was “emotional burden” (Table 2). Table 3 presents the demographic and clinical variables associated with one or more B-DDS domains. “Emotional burden”, “regimen-related distress”, and “interpersonal distress” were related to use of insulin therapy (p=0.0010), marital status (p=0.0110) and presence of three or more comorbidities (p=0.0175), respectively. The total B-DDS score was associated with marital status (p=0.0230), use of diet and physical activity to manage the disease (p=0.0180) and use of insulin therapy (p=0.0030).

**Table 2:** Average score of Brazilian version of the Diabetes Distress Scale

B-DDS	Mean (SD)
Emotional burden	3.2 (1.45)
Physician-related distress	2.0 (1.35)
Regimen-related distress	2.5 (1.38)
Interpersonal distress	2.0 (1.41)
Total score	2.6 (1.07)

B-DDS: Brazilian version of the Diabetes Distress Scale; SD: standard deviation.

**Table 3:** Factors associated with diabetes-related distress

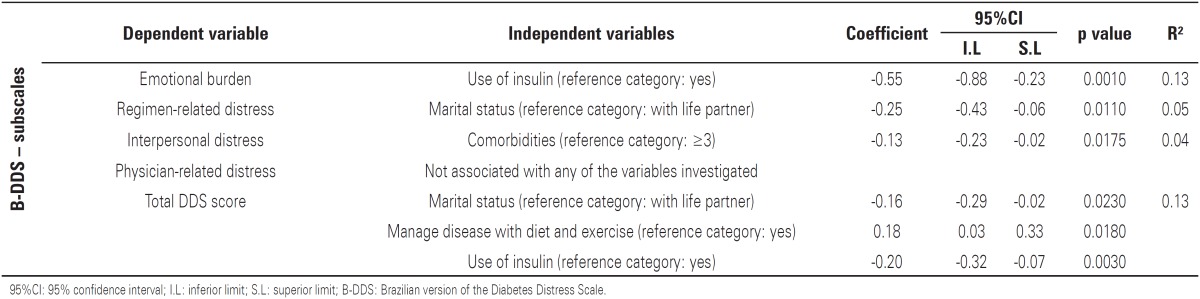

## DISCUSSION

To the best of our knowledge, this is the first study to use the B-DDS in a clinical context, in an effort to provide new information about the relation between diabetes-related distress and various social, demographic, and clinical variables in DM2 patients. Our sample consisted of patients attending a tertiary care outpatient center. Most participants were senior citizens, lived with a partner, had limited education and a low income, and had been diagnosed with DM2 more than 5 years before the study.

The prevalence of diabetes-related distress in the sample was 31.5%, which is within the range of rates reported in other studies (10 to 45%).^(1,4,12,13)^ We found that use of insulin therapy was correlated with the score on the emotional burden subscale of the B-DDS, but the results from the linear regression analyses showed that its variables were very weak predictors of diabetes-related distress (R^2^=0.13). A prior study found that the need to begin insulin therapy had very negative connotations for people with DM2.^(25)^

Patients who use insulin require frequent monitoring of capillary glycemia, food fractioning may restrict their daily activities, and management of these factors demands that the individual's complex competence level be good. Changes in emotional distress may be better explained by changes in subjective variables such as coping style and perceived support than by changes in clinical characteristics.^(26)^

Regimen-related distress was significantly associated with living with a life partner, but proved a weak predictor of diabetes-related distress. The presence of a life partner may influence the daily self-care regime of patients, as they may be subjected to more supervision and be warned frequently by their partner about the need to pay attention to their disease, thus generating higher levels of diabetes-related distress.^(27)^ It is also possible that partners may deal with the DM2 patient's health condition in a more caring way, and try to hide or disguise their concerns about the progress of the patient's disease and his or her condition. This strategy could have a negative impact on self-management of the disease.^(28)^

The presence of three or more comorbidities was significantly associated with interpersonal distress in DM2 patients; nonetheless, this was a weak predictor of diabetes-related distress. The presence of comorbidities, which are very common, was negatively associated with quality of life in patients with DM2.^(29)^ Another study reported that diabetes-related distress as measured with the DDS was negatively associated with quality of life.^(30)^

Although the independent variables “insulin treatment”, “living with a life partner”, and “presence of three or more comorbidities” were not strong predictors of scores on any of the B-DDS subscales, the evaluation of diabetes-related distress showed high distress prevalence in people with DM2, corroborating the previous studies.^(1,4,12,13)^

Additionally, we have found that most patients are overweight or obese and that HbA1C was higher than >7.0%, even though most patients self-report as following a diet and engaging in physical exercise to manage disease. Our results suggest that compliance with dietary guidelines or physical exercise is poor, and the misperception perhaps happens because patients understand that weight and physical exercise management is important for their health. Few studies have investigated associations between diabetes-related distress and self-management behaviors of diet and physical exercise; a recent study demonstrated that distress or depressive symptoms are associated with worse self-management behaviors in diabetic patients, and attention of the healthcare professionals to mental health status helps improve compliance with diet and physical exercise recommendations.^(31)^

In spite of the fact that DDS17 is not a short scale, the use of this tool contributed to screening and assessing diabetes-related emotional distress in clinical practice. Another possibility is to use DDS2 in clinical consultations, with a short form of DDS17. A study showed that the DDS2 is easier for addressing psychological issues, but this scale does not have as strong a relation with glycemia control as DDS17.^(32)^

The limitations of this study should be taken into account when interpreting the results. First, we used secondary data from previous research with a small sample. Cohort studies should be conducted in order to better explore demographic and clinical variables in diabetes-related distress. Second, the study was based on a sample of DM2 patients treated in a general adult outpatient center, and our sample may not be representative of the population of diabetics. Our participants may have been generally more severely affected by the disease than patients treated in primary care settings. Third, the study did not collect data on the incidence of chronic complications, which predict diabetes-related distress.

Our findings confirm that there is a correlation between the diabetes-related distress and DM2, which is a challenge for healthcare systems. The healthcare providers should organize to offer support and education on self-care addressing healthy lifestyle, use of medications, strategies for emotional stress and behavior change, aiming to maintain optimal metabolic control. Using specific scales to evaluate diabetes-related distress may improve overall disease management by enabling individual stress factors to be identified in a timely manner; this would enable appropriate intervention to be offered more promptly.

## CONCLUSION

Our study provided new information about the connection between diabetes-related distress and social, demographic, and clinical variables in adult type 2 *diabetes mellitus* patients. These variables are relatively weak predictors of diabetes-related distress as measured by the Diabetes Distress Scale. The mean Diabetes Distress Scale total score in our sample showed moderate patient distress, and the emotional burden domain had the highest scores (which indicate greater distress). Health professionals should put forth more effort to identify patients in whom type 2 *diabetes mellitus* has a negative psychological impact, help them with the self-management of their disease, and refer them to programs that can offer the support necessary.

## References

[1] American Diabetes Association (2014). Standards of medical care in diabetes--2014. Diabetes Care.

[2] Png ME, Yoong JS (2014). Evaluating the cost-effectiveness of lifestyle modification versus metformin therapy for the prevention of diabetes in Singapore. PLoS One.

[3] Guariguata L, Whiting DR, Hambleton I, Beagley J, Linnenkamp U, Shaw JE (2014). Global estimates of diabetes prevalence for 2013 and projections for 2035. Diabetes Res Clin Pract.

[4] Hlatky MA, Chung SC, Escobedo J, Hillegass WB, Melsop K, Rogers W, Brooks MM, BARI 2D Study Group (2010). The effect of obesity on quality of life in patients with diabetes and coronary artery disease. Am Heart J.

[5] Schram MT, Baan CA, Pouwer F (2009). Depression and quality of life in patients with diabetes: a systematic review from the European depression in diabetes (EDID) research consortium. Curr Diabetes Rev.

[6] Snoek FJ, Bremmer MA, Hermanns N (2015). Constructs of depression and distress in diabetes: time for an appraisal. Lancet Diabetes Endocrinol.

[7] Polonsky WH, Fisher L, Earles J, Dudl RJ, Lees J, Mullan J (2005). Assessing psychosocial distress in diabetes: development of the diabetes distress scle. Diabetes Care.

[8] Ridner SH (2004). Psychological distress: concept analysis. J Adv Nurs.

[9] Fisher L, Hessler DM, Polonsky WH, Mullan J (2012). When is diabetes distress clinically meaningful?: establishing cut points for the Diabetes Distress Scale. Diabetes Care.

[10] Tabák AG, Akbaraly TN, Batty GD, Kivimäki M (2014). Depression and type 2 diabetes: a causal association?. Lancet Diabetes Endocrinol.

[11] Gonzalez JS, Shreck E, Psaros C, Safren SA (2015). Distress and type 2 diabetes-treatment adherence: a mediating role for perceived control. Health Psychol.

[12] Snoek FJ, Kersch NY, Eldrup E, Harman-Boehm I, Hermanns N, Kokoszka A (2011). Monitoring of Individual Needs in Diabetes (MIND): baseline data from the Cross-National Diabetes Attitudes, Wishes, and Needs (DAWN) MIND study. Diabetes Care.

[13] Pouwer F, Wijnhoven HA, Ujcic-Voortman JK, de Wit M, Schram MT, Baan CA (2013). Ethnic aspects of emotional distress in patients with diabetes--the Amsterdam Health Monitor Study. Diabet Med.

[14] Fisher L, Mullan JT, Arean P, Glasgow RE, Hessler D, Masharani U (2010). Diabetes distress but not clinical depression or depressive symptoms is associated with glycemic control in both cross sectional and longitudinal analyses. Diabetes Care.

[15] Gonzalez JS, Delahanty LM, Safren SA, Meigs JB, Grant RW (2008). Differentiating symptoms of depression from diabetes-specific distress: relationships with self-care in type 2 diabetes. Diabetologia.

[16] Lin EH, Rutter CM, Katon W, Heckbert SR, Ciechanowski P, Oliver MM (2010). Depression and advanced complications of diabetes: a prospective cohort study. Diabetes Care.

[17] Hermanns N, Schmitt A, Gahr A, Herder C, Nowotny B, Roden M (2015). The effect of a diabetes-specific cognitive behavioral treatment program (DIAMOS) for patients with diabetes and subclinical depression: results of a randomized controlled trial. Diabetes Care.

[18] Anderson RJ, Grigsby AB, Freedland KE, de Groot M, McGill JB, Clouse RE (2002). Anxiety and poor glycemic control: a meta-analytic review of the literature. Int J Psychiatry Med.

[19] Delahanty LM, Grant RW, Wittenberg E, Bosch JL, Wexler DJ, Cagliero E (2007). Association of diabetes-related emotional distress with diabetes treatment in primary care patients with type 2 diabetes. Diabet Med.

[20] Kovacs Burns K, Nicolucci A, Holt RI, Willaing I, Hermanns N, Kalra S, Wens J, Pouwer F, Skovlund SE, Peyrot M, DAWN2 Study Group (2013). Diabetes Attitudes, Wishes and Needs second study (DAWN2TM): cross-national benchmarking indicators for family members living with people with diabetes. Diabet Med.

[21] Curcio R, Alexandre NM, Torres HC, Lima MH (2012). Tradução e adaptação do “Diabetes Distress Scale - DDS” na cultura brasileira. Acta Paul Enferm.

[22] Apolinario PP, Trevisan DD, Rodrigues RC, Jannuzzi FF, Ferreira JF, de Oliveira HC (2016). Psychometric Performance of the Brazilian Version of the Diabetes Distress Scale (B-DDS) in Patients With Diabetes Mellitus type 2. J Nurs Meas.

[23] Montgomery DC, Peck EA, Vining GG (1982). Introduction to linear regression analysis.

[24] Cohen J (1988). Statistical power analysis for the behavioral sciences.

[25] Wang HF, Yeh MC (2012). Psychological resistance to insulin therapy in adults with type 2 diabetes: mixed-method systematic review. J Adv Nurs.

[26] Karlsen B, Oftedal B, Bru E (2012). The relationship between clinical indicators, coping styles perceived support and diabetes-related distress among adults with type 2 diabetes. J Adv Nurs.

[27] Rook KS, August KJ, Choi S, Franks MM, Stephens MA (2015). Emotional reactivity to daily distress, spousal emotional support, and fasting blood glucose among patients with type 2 diabetes. J Heath Psychol.

[28] Johnson MD, Anderson JR, Walker A, Wilcox A, Lewis VL, Robbins DC (2014). Spousal protective buffering and type 2 diabetes outcomes. Health Psychol.

[29] Adriaanse MC, Drewes HW, van der Heide I, Struijs JN, Baan CA (2016). The impact of comorbid chronic conditions on quality of life in type 2 diabetes patients. Qual Life Res.

[30] Schmitt A, Reimer A, Kulzer B, Haak T, Ehrmann D, Hermanns N (2015). Research: educational and psychological aspects how to assess diabetes distress: comparison of the Problem Areas in Diabetes Scale (PAID) and the Diabetes Distress Scale (DDS). Diabet Med.

[31] Johnson ST, Al Sayah F, Mathe N, Johnson JA (2016). The relationship of diabetes-related distress and depressive symptoms with physical activity and dietary behaviors in adults with physical activity and dietary behaviors in adults with type 2 diabetes: a cross-sectional study. J Diabetes Complications.

[32] Johansen CB, Torenholt R, Hommel E, Wittrup M, Willaing I (2014). A consultation dialogue tool helps address psychological aspects of diabetes. Diabet Med.

